# Coil Migration After Embolization of Hepatic Artery Pseudoaneurysm Due to Biliary Stent Erosion

**DOI:** 10.7759/cureus.85877

**Published:** 2025-06-12

**Authors:** Carter E Edmunds, Lindsay Duy, Clancy J Clark, Rishi Pawa, Swati Pawa

**Affiliations:** 1 Internal Medicine, Wake Forest University School of Medicine, Winston-Salem, USA; 2 Radiology, Atrium Health Wake Forest Baptist Medical Center, Winston-Salem, USA; 3 Surgery, Atrium Health Wake Forest Baptist Medical Center, Winston-Salem, USA; 4 Gastroenterology, Atrium Health Wake Forest Baptist Medical Center, Winston-Salem, USA

**Keywords:** biliary stenting, coil embolization, coil migration, endoscopic retrograde cholangiopancreatography, pseudoaneurysm, transcatheter arterial embolization

## Abstract

Coil embolization migration is a rare complication of transcatheter arterial embolization intervention. This report describes a 69-year-old male who experienced massive hematemesis due to an iatrogenic hepatic artery pseudoaneurysm caused by erosion from a metal biliary stent. His pseudoaneurysm was treated by coil embolization, initially with resolution of bleeding, but was later complicated by intra-abdominal infections in the setting of the coil migration into the gastrointestinal tract. Consequently, the coil was removed endoscopically with resolution of symptoms. Currently, there are no standardized guidelines for managing migrated coils; however, symptomatic cases often require intervention due to the risk of bleeding, perforation, or infection if not addressed. This case highlights the importance of monitoring patients after undergoing transcatheter arterial embolization and considering endoscopic removal as a practical management consideration.

## Introduction

Transcatheter arterial embolization (TAE) has become an increasingly valuable option for treating and preventing gastrointestinal (GI) bleeding arising from arterial aneurysms, pseudoaneurysms, and GI ulcers [1,2]. TAE commonly uses coils to promote thrombosis and cause blood flow occlusion within the vessel to control bleeding or prophylactically prevent bleeding [3]. Despite its high clinical success rate, coil embolization can be complicated by bleeding, infection, infarction of structures supplied by the vessel or contiguous vessels, or coil migration [1,2,4-6].

While coil migration into the GI tract is extremely rare, it remains a potentially dangerous complication of this treatment. Historically, embolization coils to visceral arteries migrated frequently to the stomach; however, other studies demonstrate that the coils have also been shown to migrate to the common bile duct, small bowel, colon, and rectum within the GI tract [1,4,7,8]. Migration can lead to numerous clinical complications, including bleeding, perforation, or infection [1,4,9]. This study describes a rare instance of coil migration into the jejunal lumen after TAE of a right hepatic artery (RHA) pseudoaneurysm that was successfully managed with endoscopic intervention.

## Case presentation

**Figure 1:**
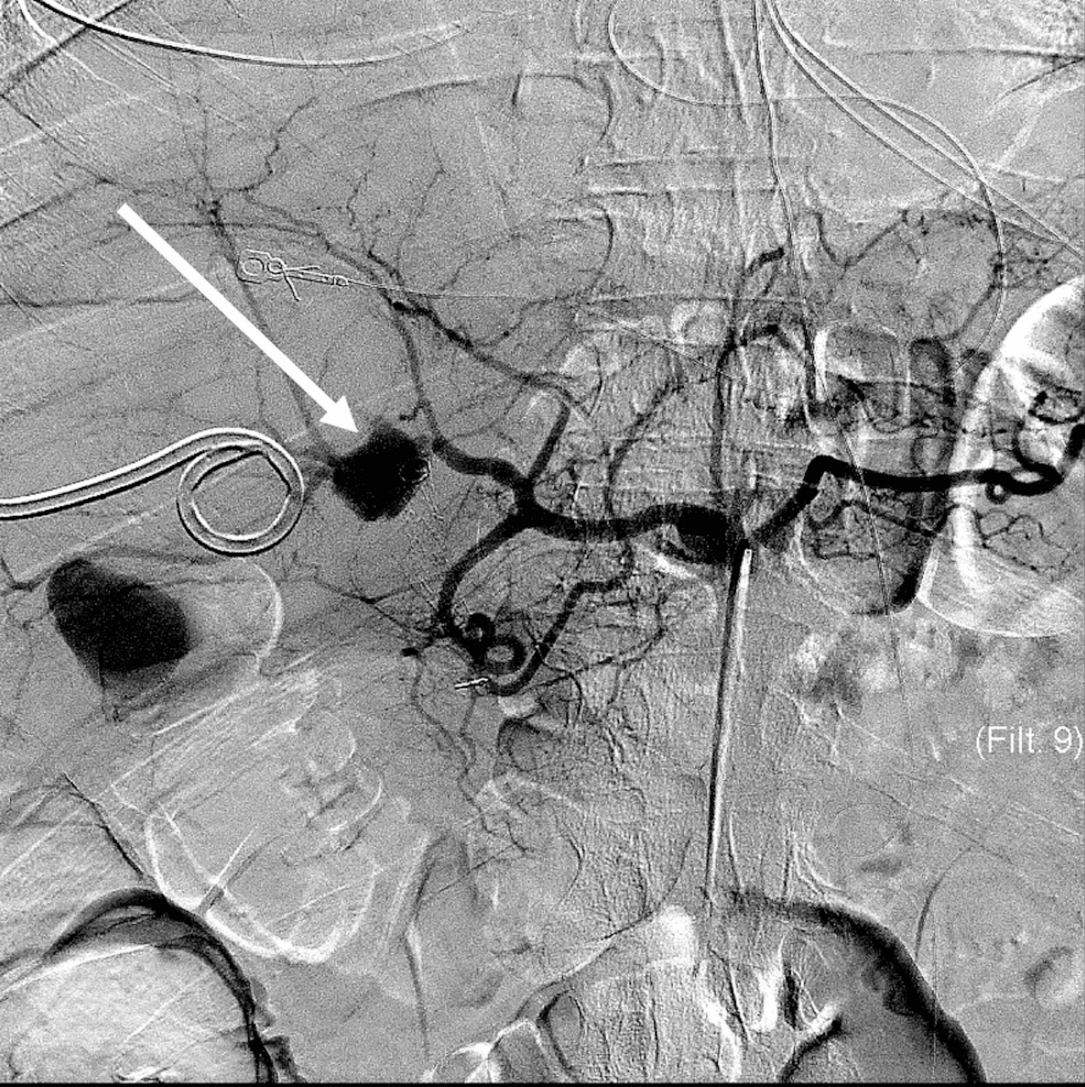
Arteriogram of the common hepatic artery showing the pseudoaneurysm arising from the right hepatic artery (arrow).

**Figure 2:**
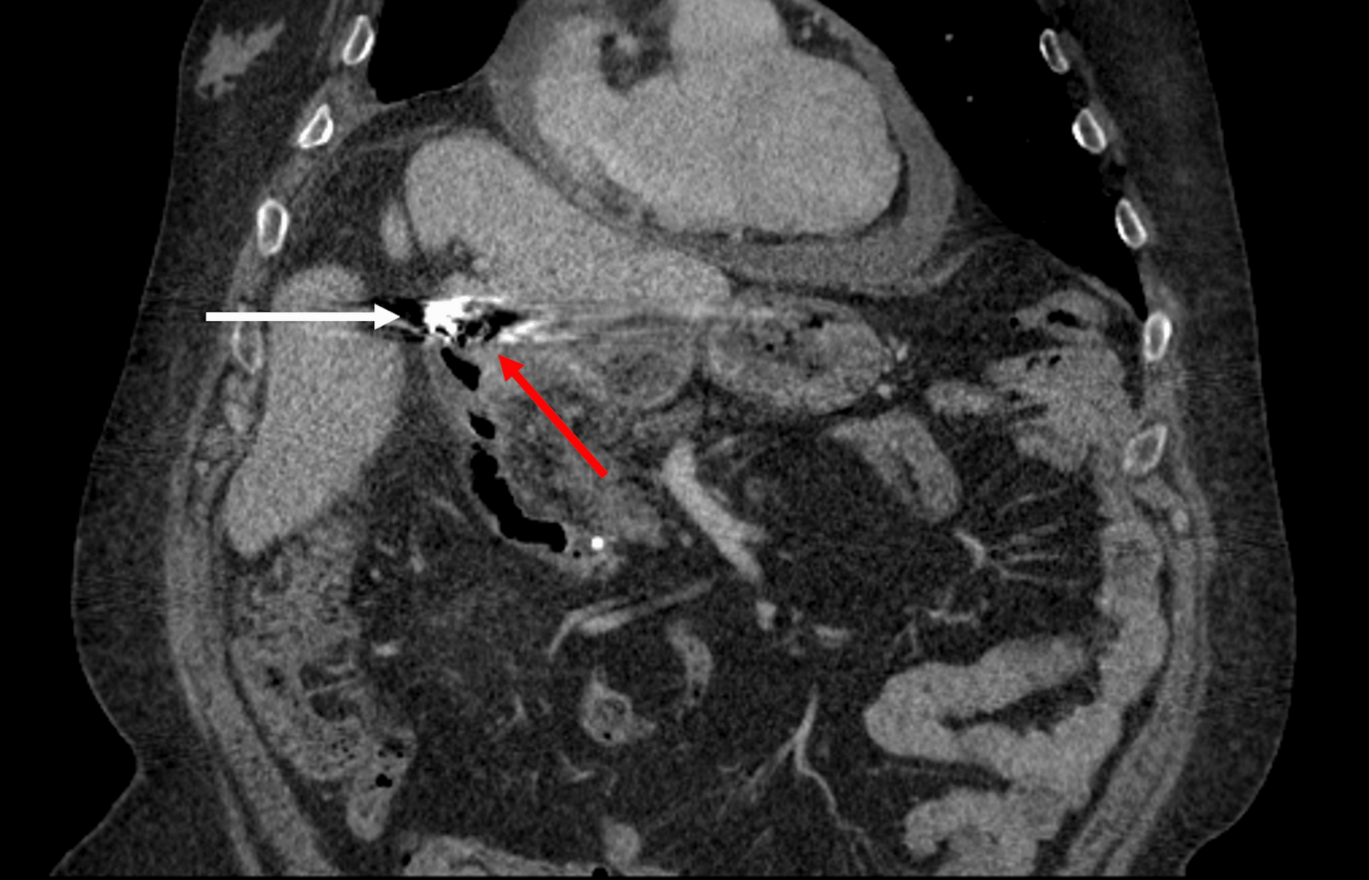
Coronal CT immediately after the transcatheter arterial embolization procedure showing the embolization coils (white arrow) in close proximity to the duodenal bulb (red arrow).

A 69-year-old male underwent open cholecystectomy for acute gangrenous cholecystitis that was complicated by a high-grade bile leak requiring endoscopic retrograde cholangiopancreatography (ERCP) with metal stent placement at another hospital. He subsequently presented to our emergency department with massive hematemesis, which was identified on imaging at arrival as likely secondary to an iatrogenic RHA pseudoaneurysm, caused by distal migration of the metal stent and subsequent erosion of the hepatic artery (Figure 1). The patient underwent successful RHA coil embolization by interventional radiology. After the procedure, a computed tomography (CT) scan showed the embolization coils appropriately placed within the RHA pseudoaneurysm near the duodenal bulb (Figure 2). Additionally, due to persistent bile leak and choledocholithiasis, an ERCP was performed to place a plastic biliary stent in the extrahepatic bile duct proximal to the metal stent. Subsequent ERCPs were performed, which led to resolution of the bile leak and choledocholithiasis; therefore, the plastic stent and metal stent were then removed. Although the patient improved after the interventions, his clinical course was complicated by a liver abscess and recurrent intra-abdominal infections, requiring multiple prolonged antibiotic courses as an outpatient.

**Figure 3:**
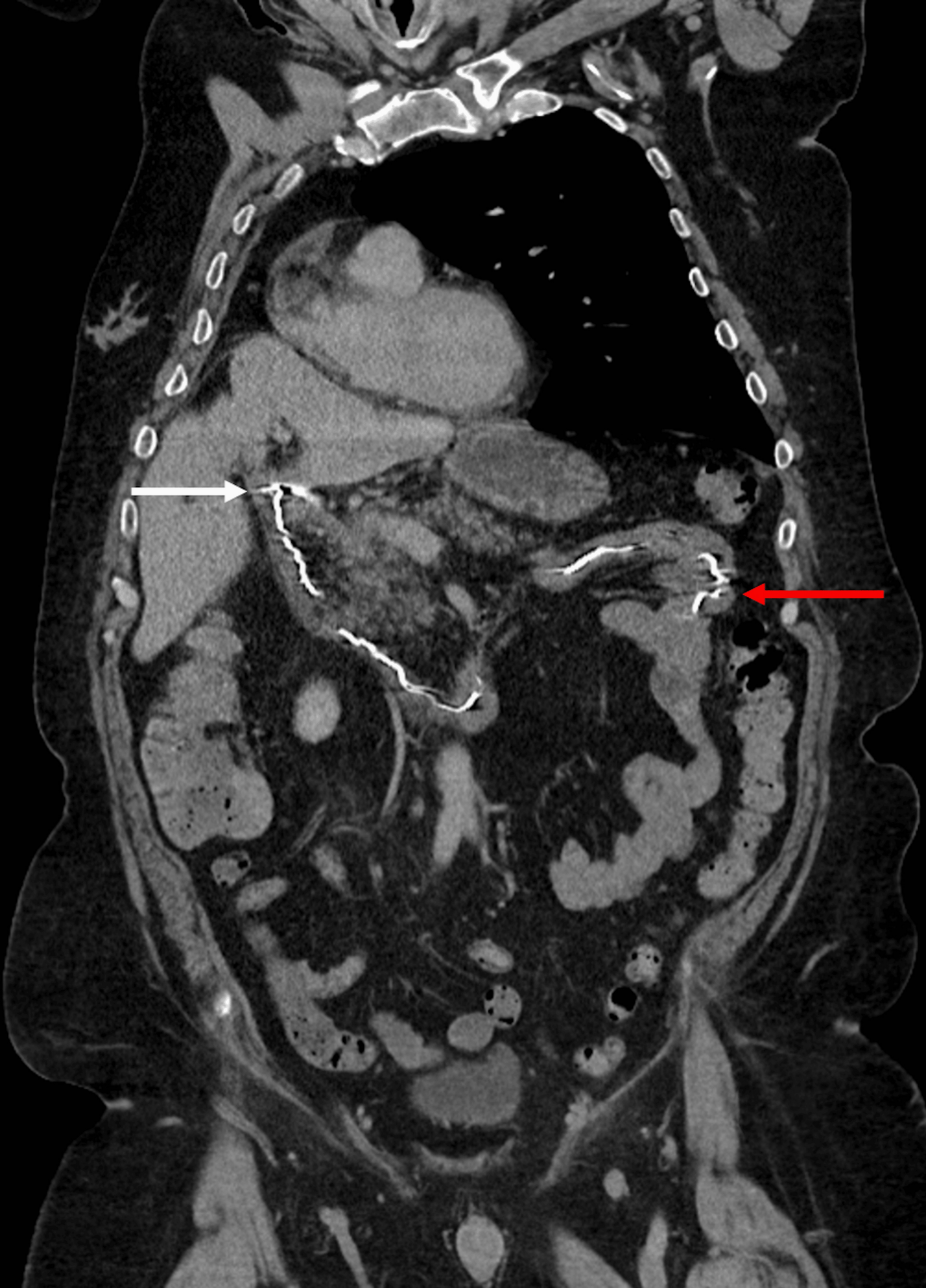
Embolization coil is seen uncoiled within the gastrointestinal tract. The white arrow shows the coil starting at the duodenal bulb and extending continuously to the jejunum, noted by the red arrow.

**Figure 4:**
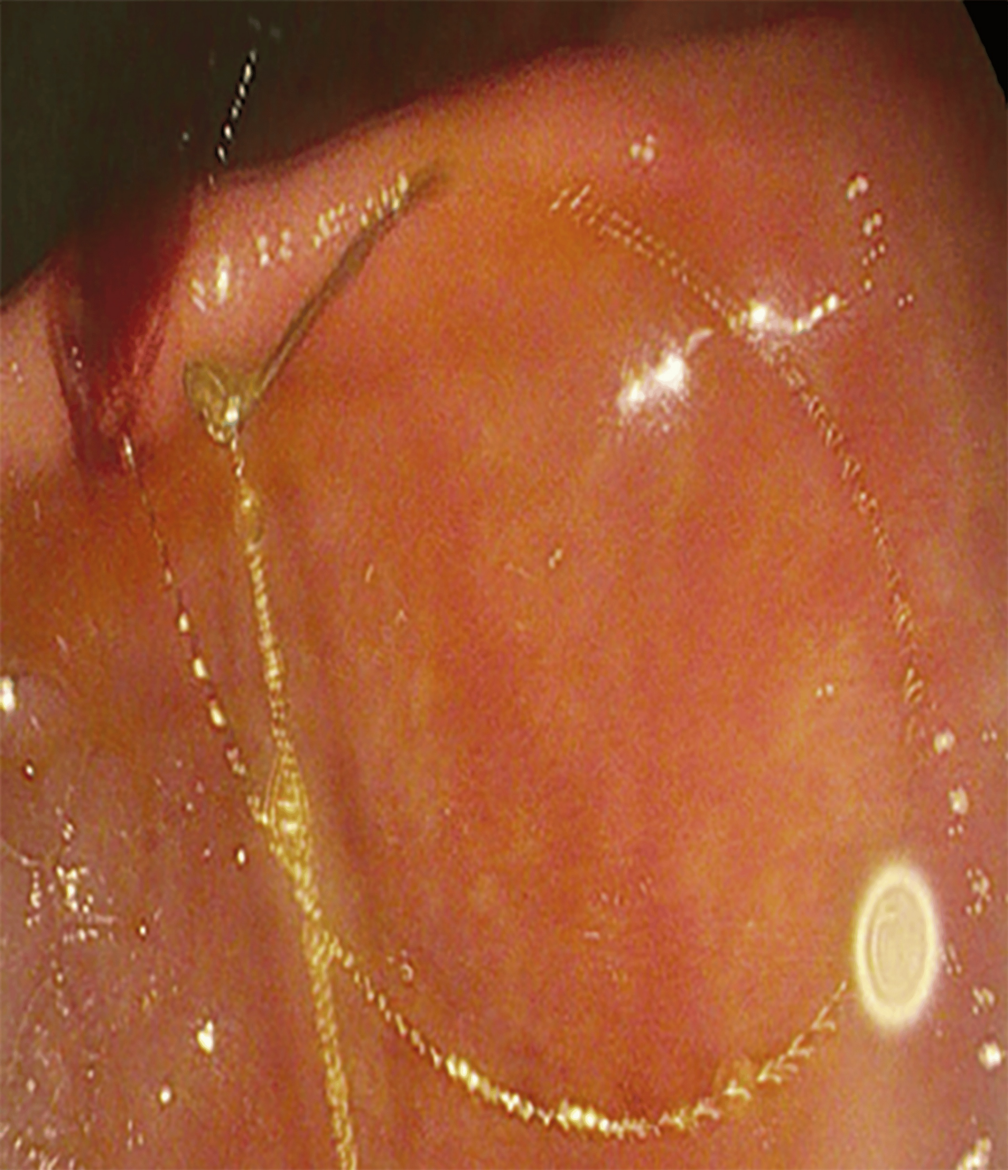
Upper endoscopy following initial CT scan confirming the unraveled coil in the duodenum.

**Figure 5:**
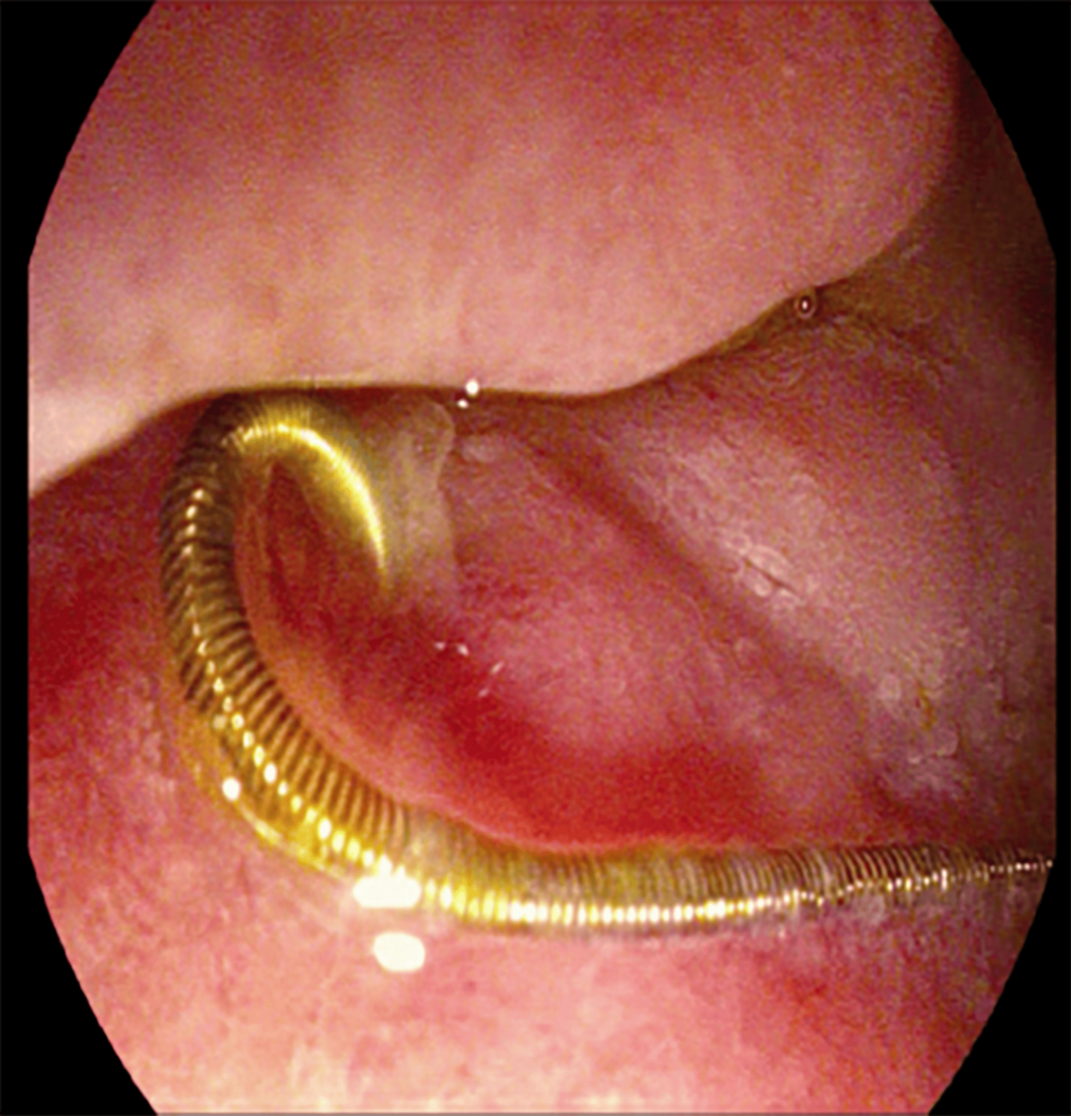
Endoscopic visualization of the coil arising from the previously treated right hepatic artery pseudoaneurysm and entering the duodenal bulb via a fistula.

Two years later, the patient presented again due to a recurrence of abdominal pain, bloating, fever, and new weight loss, concerning for a recurrent intra-abdominal infection. A CT scan showed a migrated endovascular coil in the duodenal wall extending to the jejunum (Figure 3). An esophagogastroduodenoscopy was then performed to determine if the unraveled coil could be easily located and potentially manually removed, preventing the patient from needing a more invasive surgical intervention. The upper endoscopy confirmed the presence of the unraveled coil extending into the duodenal lumen that continued distally and was able to be separated from the bowel wall without resistance (Figure 4). The coil was grasped with rat tooth forceps and 45 cm of the coil was removed. The point of fistula formation between the duodenal bulb and previously treated RHA pseudoaneurysm was seen in the duodenal sweep and was confirmed under fluoroscopic imaging (Figure 5). An additional 10 cm of coil wire at the site of the fistula was trimmed with endoscissors and removed. The patient had no residual symptoms or episodes of intra-abdominal infections and remained well at his six-month follow-up.

## Discussion

Over recent decades, TAE has become an ideal management option for visceral artery aneurysms and pseudoaneurysms. In some studies, it offers a minimally invasive alternative with a high success rate [5,6,10-13]. Despite its high success rate and safety profile, complications, such as ischemia, infection, bleeding, nontarget embolization, and coil migration, can occur [1,2,4-6].

Coil migration from TAE is an infrequent phenomenon that can occur within a few weeks to even years from the initial procedure and has a broad spectrum of presentations. Very few cases have been published thus far. Most documented cases involve coil migration into the stomach, with duodenal involvement being significantly more rare [1]. Coil migration into the gastrointestinal tract is often asymptomatic, but this complication can also potentially lead to severe bleeding, perforation, or infection [1,7].

In prior case reports, it has been suggested that conservative management could be sufficient if the patient is asymptomatic from the migration; however, this still carries the potential risk of delayed complications [1,7,9]. Multiple previously published cases with asymptomatic coil migration to the stomach were managed conservatively without consequences [1]. There are reported cases of asymptomatic coil migration to the sigmoid colon or rectum that were also successfully managed conservatively [7,14]. However, complications of bleeding or perforation have been reported in prior cases, which frequently necessitated further intervention. For instance, Chang et al. described a case with a coil migration to the stomach causing a continuous bleeding gastric ulcer, which had to be treated endoscopically [2]. In rare cases, such as that reported by Dinter et al., coil migration to the gastric cardia led to a lethal aortogastric fistula a decade following the initial embolization [9]. In certain cases, endoscopic removal was attempted to manage the coil migration. Han et al. described a case with successful retrieval of the migrated coil into the stomach via endoscopy [4].

In contrast to the previously reported cases, the present case is unique in that the overall size of the coil within the gastrointestinal tract was significant, and the removal technique used during the procedure required a multi-step process. A dual-stage extraction technique was utilized first to remove the distal 45 cm of the migrated coil that extended into the jejunum. Once this portion of the coil was removed, the proximal 10 cm remaining coil that extended from the fistula site was then cut to be flush with the duodenal wall and removed. Additionally, the location of the coil migration to the duodenum is rare, given that many prior cases have described migration to the stomach.

While the mechanisms that lead to coil migration are not fully understood at this time, factors that can increase the occurrence are thought to be the artery location and method of embolization, vessel anatomy, and presence of collaterals, or local underlying inflammation and structure breakdown [1,5]. In previous cases and our case, it is unclear if the localized infection was a nidus for the nearby structure breakdown and subsequent migration versus if the coil led to the nearby structure breakdown and acted as the nidus for infection. In some previous case reports, the migration was preceded by an inflammatory event and believed to incite the migration; however, in other studies, there was no inciting inflammatory event preceding the migration [7]. Unfortunately, given the limited cases, it is difficult to determine which mechanism is the true driving force. Regardless of the etiology of the coil migration, there are different approaches to treating this complication. Due to the infrequency of coil migration, there are currently no standardized management guidelines.

When determining if conservative management without intervention, surgical removal, or endoscopic removal should be chosen, it is important to consider the patient’s current presentation in the decision process. While some clinicians in the past have chosen to be more proactive, despite the lack of significant symptoms, out of an abundance of caution, this may not be warranted, given some prior cases have shown spontaneous excretion of the coil. However, if the migrated coil continues to cause complications, multiple cases have been successfully intervened on using a surgical or endoscopic approach.

Our experience with endoscopic removal of the migrated coil, as described in the case herein, demonstrates that endoscopic intervention can be used as a less invasive approach that is feasible and effective. This technique may be more advantageous in high-risk surgical patients or if the coil appears to be accessible via endoscopy. This case and others bring to light the broader implications of ways to manage migrations from TAE. All patients undergoing TAE need to be closely monitored for signs of coil migration and sequelae, but not all patients need intervention, as shown in other cases. There is currently no recommended timeline for follow-up when asymptomatic migration occurs, given the rarity of this phenomenon. Patients typically are counseled on symptom monitoring for possible complications of TAE after the initial procedure, which is adequate for the majority of cases.

## Conclusions

In conclusion, this study highlights an uncommon yet clinically significant complication of TAE. While TAE remains a highly effective and minimally invasive intervention for managing visceral artery aneurysms and pseudoaneurysms, clinicians should be aware of delayed complications such as coil migration, which may present asymptomatically or with life-threatening consequences. As demonstrated in the present study, endoscopic retrieval offers a safe and feasible alternative to surgery for migrated coils, particularly in patients deemed high surgical risk. Given the lack of standardized management guidelines, clinical decision-making should be guided by the patient's clinical presentation, risk of complications, and endoscopic or surgical removal feasibility. As coil migration becomes more prevalent in the setting of increased TAE procedures over time, future research will be crucial for identifying additional risk factors for coil migration, improving embolization techniques to prevent migration, and establishing standardized guidelines for surveillance and management of coil migration.
